# Trends in the use and dual use of factory-made combustible cigarettes, other tobacco products and electronic cigarettes: Results from South African Social Attitudes Surveys during 2007 to 2018

**DOI:** 10.18332/tid/168121

**Published:** 2023-07-17

**Authors:** Catherine O. Egbe, Siphesihle Gwambe, Mukhethwa Londani, Olufemi Erinoso, Olalekan A. Ayo-Yusuf

**Affiliations:** 1 Alcohol, Tobacco and Other Drug Research Unit, South African Medical Research Council, Pretoria, South Africa; 2 Department of Public Health, Sefako Makgatho Health Sciences University, Pretoria, South Africa; 3 Directorate of Research and Innovation, Tshwane University of Technology, Pretoria, South Africa; 4 School of Public Health, University of Nevada, Reno, United States; 5 Africa Centre for Tobacco Industry Monitoring and Policy Research, School of Health System and Public Health, University of Pretoria, Pretoria, South Africa

**Keywords:** tobacco use, dual product use, combustible tobacco, e-cigarettes, South Africa

## Abstract

**INTRODUCTION:**

Using more than one tobacco product increases the risk of tobacco-related diseases. We investigated trends in the prevalence and dual use of factory-made (FM) cigarettes, other tobacco products, and electronic cigarettes (e-cigarettes) in South Africa over a 12-year period.

**METHODS:**

Data from five waves (2007, 2010, 2011, 2017, and 2018) of the South African Social Attitudes Survey (n=14582) were analyzed. The use of FM, roll-your-own (RYO) cigarettes, cigars, waterpipe, smokeless tobacco (SLT), any combustible tobacco products (CTP), any tobacco product (ATP) use, and e-cigarettes was investigated. The dual use of FM cigarettes with either SLT, waterpipe or e-cigarettes was also explored. Chi-squared analyses and regression models were used to explore trends in prevalence over the 12-year period.

**RESULTS:**

About 51% of the participants were female, and 51.9% were aged 16–34 years. CTP smoking significantly increased from 18.1% (2010) to 23.6% (2018) (p=0.015), while ATPU increased from 20.2% (2010) to 25.9% (2018) (p=0.005). Though dual use of FM cigarettes and SLT, waterpipe, or e-cigarettes was generally low, the prevalence of dual use significantly increased for all product combinations investigated: FM cigarettes and SLT (0.5% in 2007 to 1.3% in 2018, p=0.017), FM cigarettes and waterpipe (0.9% in 2010 to 2.5% in 2018, p=0.014), FM cigarettes and e-cigarettes (0.4% in 2010 to 1.8% in 2018, p<0.001). Compared to 2010, the odds of the prevalence of CTP and ATP use significantly increased by 37% in 2018 (adjusted odds ratio, AOR=1.37; 95% CI: 1.06–1.77; p=0.018 and AOR=1.37; 95% CI: 1.08–1.73; p=0.009, respectively) during the 12-year period after adjusting for demographic characteristics.

**CONCLUSIONS:**

The use and dual use of tobacco and electronic cigarette products have been increasing in recent years in South Africa. Interventions to help users quit and prevent young people from initiating use are urgently needed to curb these increases.

## INTRODUCTION

Tobacco use, especially combustible cigarettes, is implicated in the death of more than 8 million people annually worldwide, and most of these deaths occur in low- and middle-income countries (LMICs)^1,2^. The implementation of the WHO Framework Convention on Tobacco Control (WHO FCTC) has been identified as one of the sustainable development goals for promoting population health and well-being^3^. Despite some progress in the implementation of the WHO FCTC and the reduction in smoking prevalence observed in several parts of the world^3^, there are still gaps in tobacco control, including the promotion of new or so-called ‘alternative’ tobacco or nicotine products that may undermine current efforts to control cigarette smoking^4^.

A growing trend of dual-use or poly-use of tobacco/nicotine products has emerged globally, involving combined or concurrent use of combustible cigarettes and smokeless tobacco, with novel nicotine products such as e-cigarettes or re-emerging tobacco products such as waterpipe^5,6^. The concurrent use of combustible tobacco products with other tobacco/nicotine products presents a public health problem^5^, as it can potentially prolong and sustain nicotine dependence and exposure to more toxicants^7^. The adverse impact of dual tobacco and nicotine product use on health includes elevated risk of cardiovascular and pulmonary diseases^8-10^. In addition, the dual use of these products may encourage smokers to defer cessation; therefore, dual users are less likely to intend to quit than those who use only one tobacco or nicotine product^11^. Dual users emerged as a high-risk group for nicotine dependence and tobacco-related harm^7,11,12^.

In South Africa, about 7% of all deaths are attributed to smoking but this increases to about 17% for people aged ≥35 years^13^. Results from the 2016 South African Demographic and Health Survey show that 37% of men and 6.8% of women aged ≥15 years use at least one tobacco product^14^. Previous studies have reported a significant reduction in the prevalence of tobacco use in South Africa (SA)^15,16^, following the introduction of the Tobacco Products Control Act 83 of 1993 (amended 2008)^17^. However, this reduction in prevalence has stabilized with no change in quitting behavior over the same period^18^, and recent increases in tobacco use have been reported between 2008 and 2011 among South Africans^13,19^. The re-bound and increase in tobacco use prevalence have been attributed to the marketing and social acceptance of novel and re-emerging tobacco/nicotine products such as e-cigarettes and waterpipe tobacco which come in exotic flavors, and promoted for use in social settings^6^. Limited data are available on the trends of dual tobacco/nicotine product use among the South African population. Continuous surveillance to monitor patterns of use of tobacco/nicotine products in the population is crucial to evaluation and the assessment of a country’s population health and public health programs and policies. Therefore, this study investigates trends in the prevalence of use of tobacco and nicotine products, including factory-manufactured (FM) cigarettes, roll-your-own (RYO) cigarettes, cigars, waterpipe tobacco, smokeless tobacco (SLT) (oral and nasal snuff), and electronic cigarettes (e-cigarettes), as well as dual use of FM cigarettes with either a waterpipe, SLT, or e-cigarettes, for people aged ≥16 years in South Africa over a 12-year period using the South African Social Attitude Survey (SASAS).

## METHODS

### Research design

This study utilized five waves (2007, 2010, 2011, 2017, and 2018) of the South African Social Attitudes Survey (SASAS) conducted among individuals aged ≥16 years in South Africa. SASAS is a cross-sectional national household survey used to monitor the general public’s evolving social, economic, and political values and behaviors since 2003 and conducted by the Human Sciences Research Council (HSRC)^20^. The survey samples were drawn from the HSRC master sample frame, consisting of 1000 population census enumeration areas (EAs) demarcated for Census 2011^21^. These EAs served as primary sampling units in the multi-stage probability sampling strategy used for this survey. The enumeration areas were stratified by sociodemographic domains such as geographical subtype, province, and population groups to yield nationally representative samples of adults aged ≥16 years^20^. Sample weights were calculated and used when conducting analyses, taking account of response patterns so as to ensure that the sample is representative of the national population.

### Demographic characteristics

Demographic characteristics of participants evaluated include race [Black African (indigenous African descent), White, Indian/Asian and Colored (mixed ancestry)], gender (male/female), marital status (married, widowed/divorced/separated and never married) and education level (<12; 12; >12 years of schooling). Total pooled sample comprised 14582 respondents aged ≥16 years (Table 1).

**Table 1:** Demographic characteristics of all participants, South Africa, 2007–2018

*Characteristics*	*2007*		*2010*		*2011*		*2017*		*2018*		*Total*		
	*n (%)*	*95% CI*	*n (%)*	*95% CI*	*n (%)*	*95% CI*	*n (%)*	*95% CI*	*n (%)*	*95% CI*	*n (%)*	*95% CI*	*p*
**Gender**													0.203
Female	1686 (51.3)	48.5–54.2	1844 (52.2)	49.6–54.8	1763 (52.5)	50–55	1864 (51.8)	48.5–55	1614 (48.3)	45.4–51.2	8771 (51.1)	49.8–52.4	
Male	1221 (48.7)	45.8–51.5	1268 (47.8)	45.2–50.4	1240 (47.5)	45–50	1199 (48.3)	45–51.5	1122 (51.7)	48.8–54.6	6050 (48.9)	47.6–50.2	
**Race/ethnicity**													0.991
African	1812 (76.7)	72.9–80.1	1781 (76.6)	72.9–79.9	1883 (76.7)	73.2–79.9	1872 (78.5)	74.6–81.9	1724 (78.7)	74.5–82.5	9072 (77.5)	75.8–79.2	
Colored	434 (9.4)	7.3–11.9	564 (9.3)	7.5–11.6	473 (9.5)	7.6–11.9	495 (9)	7.1–11.4	409 (9.1)	6.9–11.8	2375 (9.2)	8.3–10.3	
White	335 (11.2)	8.9–14	401 (11.1)	8.9–13.7	387 (10.9)	8.8–13.4	348 (9.6)	7.4–12.5	263 (9.3)	6.8–12.7	1734 (10.4)	9.3–11.6	
Asian	326 (2.8)	2–3.7	365 (2.9)	2.2–4	259 (2.9)	2.1–3.8	348 (2.8)	2.1–3.9	337 (2.9)	2.1–3.9	1635 (2.9)	2.5–3.3	
**Marital status**													<0.001
Married	1086 (33.9)	31–36.8	1204 (33.8)	31.1–36.6	1090 (32.1)	29.6–34.8	1254 (38.6)	35.9–41.3	824 (26.8)	23.7–30.1	5458 (32.9)	31.6–34.3	
Widowed/divorced/separated	430 (12)	10.3–14	447 (10.2)	8.9–11.8	456 (10.5)	9.4–11.8	507 (10.3)	8.9–11.8	503 (11.6)	10–13.5	2343 (10.9)	10.2–11.6	
Unmarried	1378 (54.2)	51.1–57.2	1337 (56)	52.9–59.1	1374 (57.4)	54.6–60.1	1189 (51.2)	48.3–54.1	1260 (61.6)	58.1–65.1	6538 (56.2)	54.7–57.6	
**Age** (years)													0.864
16–24	686 (27.4)	25–30	618 (27.5)	24.9–30.2	566 (26.8)	24.3–29.5	498 (24.2)	21.8–26.7	433 (24)	21–27.3	2801 (25.8)	24.6–27.1	
25–34	633 (26)	23.8–28.2	699 (25.6)	23.4–28	707 (25.6)	23.5–27.8	637 (26.5)	24.2–28.9	565 (26.6)	23.9–29.6	3241 (26.1)	25–27.2	
35–44	614 (17)	15.3–18.9	656 (17.9)	16.3–19.7	568 (18.4)	16.6–20.4	613 (19)	17.4–20.8	513 (19.3)	17–21.8	2964 (18.4)	17.5–19.3	
45–54	440 (13.5)	11.8–15.4	449 (12.1)	10.5–13.8	507 (13.2)	11.7–14.8	437 (13.4)	11.7–15.4	424 (13.1)	11.4–15	2257 (13.1)	12.3–13.9	
55–64	295 (8.7)	7.4–10.2	382 (9.6)	8.2–11.3	360 (9.1)	7.8–10.5	413 (9.3)	8.1–10.7	410 (9.2)	7.9–10.6	1860 (9.2)	8.6–9.8	
≥65	231 (7.5)	6.2–9	306 (7.3)	6.1–8.7	292 (6.9)	6–8.1	465 (7.6)	6.4–9	391 (7.9)	6.6–9.5	1685 (7.5)	6.9–8.1	
**Education level** (years of schooling)													<0.001
<12	1587 (53.9)	50.5–57.2	1580 (52.9)	49.6–56.2	1559 (53.2)	50.2–56.2	1748 (56.9)	53.6–60.2	1165 (42.7)	38.8–46.6	8858 (61.2)	59.4–63	
12	638 (23)	20.6–25.5	785 (27.3)	24.8–29.8	796 (30.8)	28.2–33.5	911 (33.0)	30.2–35.8	710 (34.7)	31–38.5	2936 (22.7)	21.3–24.1	
>12	595 (23.2)	20.5–26.1	627 (19.8)	17.5–22.4	489 (16)	14–18.3	307 (10.2)	8.3–12.4	540 (22.6)	19.5–26.1	2282 (16.1)	15–17.3	

* Significant at p<0.05 (Rao-Scott chi-squared test). All data are weighted.

### Tobacco and nicotine product type

Tobacco products investigated include combustible tobacco products (FM cigarettes and RYO cigarettes, cigars, and waterpipe tobacco), as well as SLT and e-cigarettes. Data were not collected for waterpipes and e-cigarettes in 2007 as these were either not yet popular or had not been introduced into the South African market. Hence, analysis of the dual use of FM cigarettes and either waterpipes or e-cigarettes excluded 2007 data.

### Tobacco and nicotine product use

Current use included daily and non-daily use of each tobacco or nicotine product. Dual use included the use of FM cigarettes and either waterpipes, SLT, or e-cigarettes. Other dual-use categories samples were too small to run analyses. Participants who used combustible tobacco products (FM cigarettes, RYO cigarettes, cigars, and waterpipes) as well as those who used any type of tobacco product (all products excluding e-cigarettes) were also explored.

### Data analysis

Taking account of the multi-stage sampling used in SASAS, data were analyzed using Stata statistical software version 17 (StataCorp LP., College Station, TX, USA). Data were weighted to account for the complex sampling design. Frequencies were used to explore the trends in use prevalence from 2007–2018. Chi-squared tests were used to determine the association between product used and participants' race, gender, marital status, and educational level across the years with raw point estimates. Mixed effects logistic regression models were used to assess the change in prevalence of CTP and ATP use over time and for each category of the demographic characteristics (age, gender, race/ethnicity, marital status and years of schooling). Year 2010 was used as reference category for all models. The interaction effects were tested per model. All tests were 2-tailed and values of p<0.05 were considered statistically significant.

## RESULTS

### Demographic characteristics

Overall, participants comprised: 51.1% (n=8771) females; 77.5% (n=9072) Black Africans; 56.2% (n=6538) never married; and more than half aged 16–34 years (n=6042) (Table 1). Proportion of participants who were married increased from 33.9% (2007) to 38.6% (2017) and decreased to 26.8% in 2018. Similarly, proportion of those who had 12 years of schooling increased from 23% in 2007 to 34.7% in 2018.

### Prevalence of tobacco/nicotine product use by product type

*FM cigarettes*

The prevalence of those using FM cigarettes decreased from 19.3% (95% CI: 17.2–21.6) in 2007 to 16.1% (95% CI: 14.3–18.1) in 2010 and increased afterwards from 18.8% (95% CI: 16.8–21.0) in 2011 to 20.4% (95% CI: 17.8–23.4) in 2018 (Table 2).

**Table 2:** Trends in tobacco use prevalence by tobacco/nicotine product type between, South Africa, 2007–2018

*Product use*	*2007*		*2010*		*2011*		*2017*		*2018*		
	*n (%)*	*95% CI*	*n (%)*	*95% CI*	*n (%)*	*95% CI*	*n (%)*	*95% CI*	*n (%)*	*95% CI*	*p*
**Cigarette use**											**0.273**
Current	626 (19.3)	17.2–21.6	575 (16.1)	14.3–18.1	588 (18.8)	16.8–21.0	607 (19.3)	17.2–21.6	560 (20.4)	17.8–23.4	
Former	80 (2.7)	2.0–3.7	85 (2.3)	1.7–3.1	110 (3.4)	2.6–4.3	100 (3.0)	2.0–4.4	88 (3.2)	2.0–5.2	
Never	2183 (78.0)	75.6–80.3	2390 (81.6)	79.5–80.5	2254 (77.9)	75.5–80.1	2304 (77.7)	75.0–80.2	2041 (76.4)	73.1–79.3	
**Roll-your-own use**											<0.001*
Current	158 (4.2)	3.3–5.2	150 (5.0)	4.0–6.4	168 (5.3)	4.2–6.6	144 (5.3)	4.0–7.1	183 (7.9)	6.0–10.2	
Former	26 (0.7)	0.4–1.2	32 (1.4)	0.9–2.3	44 (1.4)	1.0–2.1	58 (2.6)	1.7–3.8	48 (1.3)	0.8–2.0	
Never	2686 (95.2)	94.0–96.1	2867 (93.5)	92.0–94.8	2730 (93.3)	91.9–94.6	2812 (92.2)	89.7–94.1	2458 (90.8)	88.4–92.8	
**Waterpipe use**											<0.001*
Current	NA	NA	41 (1.2)	0.8–1.9	51 (1.7)	1.1–2.5	57 (3.1)	2.1–4.5	100 (4.1)	3.0–5.7	
Former	NA	NA	29 (0.9)	0.6–1.5	22 (0.6)	0.3–1.1	49 (2.0)	1.2–3.3	61 (2.0)	1.3–2.9	
Never	NA	NA	2980 (97.9)	97.0–98.5	2867 (97.7)	96.9–98.4	2905 (94.9)	93.2–96.2	2528 (94.0)	92.1–95.4	
**Cigar use**											<0.001*
Current	21 (0.5)	0.3–0.8	32 (0.9)	0.5–1.4	33 (0.9)	0.5–1.4	40 (1.1)	0.7–1.7	57 (2.9)	1.9–4.3	
Former	9 (0.2)	0.1–0.4	23 (0.7)	0.4–1.3	27 (0.8)	0.5–1.3	30 (1.0)	0.6–1.7	46 (1.2)	0.7–2.1	
Never	2839 (99.4)	99.0–99.6	2994 (98.4)	97.7–98.9	2877 (98.3)	97.7–98.8	2944 (97.9)	97.1–98.5	2586 (95.9)	94.4–97.0	
**Combustible tobacco use†**											0.015*
Current			637 (18.1)	16.1–20.3	629 (19.8)	17.7–22.0	651 (21.1)	18.9–23.5	624 (23.6)	20.6–26.8	
Former			85 (2.4)	1.7–3.2	118 (3.7)	2.9 – 4.7	106 (3.7)	2.3– 5.9	87 (1.9)	1.3–2.7	
Never			2333 (79.5)	77.2–81.6	2207 (76.6)	74.1–78.9	2262 (75.2)	72.1–78.1	1978 (74.6)	71.3–77.6	
**E-cigarette use**											<0.001*
Current			18 (0.5)	0.2–0.9	11 (0.3)	0.1–0.6	35 (1.6)	1.0–2.6	62 (2.7)	1.7–4.4	
Former			19 (0.6)	0.3–1.3	9 (0.3)	0.1–0.6	33 (0.9)	0.6–1.4	41 (1.4)	0.9–2.2	
Never			3011 (98.9)	98.2–99.3	2917 (99.5)	99.1–99.7	2945 (97.5)	96.5–98.2	2586 (95.9)	94.1–97.2	
**Smokeless tobacco use**											0.002*
Current	117 (5.0)	3.9–6.5	89 (3.1)	2.3–4.2	78 (2.2)	1.6–2.9	130 (3.9)	3.0–5.2	110 (4.1)	3.1–5.3	
Former	13 (0.4)	0.2–0.7	27 (1.1)	0.7–1.8	19 (0.5)	0.3–0.8	31 (0.9)	0.5–1.5	27 (0.8)	0.4–1.4	
Never	2737 (94.6)	93.1–95.8	2931 (95.8)	94.5–96.8	2839 (97.3)	96.6–97.9	2850 (95.2)	93.9–96.2	2552 (95.2)	93.8–96.3	
**Cigarette and SLT use**											0.017*
Yes	11 (0.5)	0.3–1.0	16 (0.7)	0.3–1.3	17 (0.4)	0.2–0.9	17 (0.5)	0.3–0.9	29 (1.3)	0.8–2.3	
No	2878 (99.5)	99.1–99.8	3039 (99.3)	98.7–99.7	2936 (99.6)	99.2–99.8	3001 (99.5)	99.1–99.7	2660 (98.7)	0.4–1.4	
**Cigarette and waterpipe use**											0.014*
Yes			33 (0.9)	0.6–1.5	38 (1.4)	0.9–2.1	43 (2.0)	1.3–2.9	60 (2.5)	1.7–3.7	
No			3009 (99.1)	98.5–99.4	2889 (98.7)	97.9–99.2	2954 (98.0)	97.1–98.7	2589 (97.5)	96.3–98.3	
**Cigarette and e-cigarette use**											<0.001*
Yes			17 (0.4)	0.2–0.9	8 (0.2)	0.1– 0.5	29 (1.1)	0.7–1.7	41 (1.8)	1.0–3.4	
No			3031 (99.6)	99.1–99.8	2929 (99.8)	99.5–99.9	2984 (98.9)	98.3–99.3	2648 (98.2)	96.6–99.0	
											
**Any tobacco product use^‡^**											0.005*
Yes			637 (20.2)	18.3–22.4	629 (21.1)	19.0–23.4	655 (24.4)	22.1–26.8	630 (25.9)	22.8–29.3	
No			2475 (79.8)	77.6–81.8	2375 (78.9)	76.6–81.0	2408 (75.6)	73.2–77.9	2106 (74.1)	70.7–77.2	

† Combustible tobacco product use: combined cigarette, roll-your-own, cigars, pipes and waterpipe tobacco use.

‡ Any tobacco product: includes all combustible and smokeless tobacco products.

* Significant at p<0.05 (Rao-Scott chi-squared test).

*RYO cigarettes*

The prevalence of those using RYO cigarettes consistently increased from 4.2% (95% CI: 3.3–5.2) in 2007 to 7.9% (95% CI: 6.0–10.2) in 2018 (Table 2).

*Waterpipe tobacco*

No data were collected for waterpipe tobacco smoking in 2007. The prevalence of waterpipe tobacco use consistently increased from 1.2% (95% CI: 0.8–1.9) in 2010 to 4.1% (95% CI: 3.0–5.7) in 2018 (Table 2).

*Cigar*

The prevalence of cigar smoking was 0.5% in 2007 (95% CI: 0.3–0.8). Cigar smoking remained stable in 2010 and 2011. Thereafter, the prevalence increased from 1.1% (95% CI: 0.7–1.7) in 2017 to 2.9% (95% CI: 1.9–4.3) in 2018 (Table 2).

*SLT use*

The prevalence of current SLT use was 5.0% (95% CI: 3.9–6.5) in 2007, which decreased to 2.2% (95% CI: 1.6–2.9) in 2011 and afterwards increased to 4.1% (95% CI: 3.1–5.3) in 2018 (Table 2).

*E-cigarettes*

No data were collected for e-cigarette use in 2007. The prevalence of e-cigarette use was 0.5% (95% CI: 0.2–0.9) in 2010. This decreased to 0.3% (95% CI: 0.1–0.6) in 2011, then increased to 1.6% (95% CI: 1.0–2.6) in 2017 and to 2.7% (95% CI: 1.7–4.4) in 2018 (Table 2).

### Pooled prevalence of tobacco product use

*Combustible tobacco product use*

The prevalence of those using combustible tobacco products (CTP) was found to be 18.1% (95% CI: 16.1–20.3) in 2010 and increased to 19.8% in 2011 and to 23.6% (95% CI: 20.6–26.8) in 2018 (Table 2). Data for waterpipe use were not collected in 2007, hence the exclusion of 2007 from this analysis.

*Any tobacco product use*

Overall, any tobacco product (ATP) use (excluding 2007 due to lack of data for waterpipe tobacco use) showed consistent increase from 20.2% (95% CI: 18.3–22.4) in 2010 to 25.9% (95% CI: 22.8–29.3) in 2018 (Table 2).

### Prevalence of dual use of FM cigarettes and smokeless tobacco, waterpipe, or e-cigarettes

**Figure 1:**
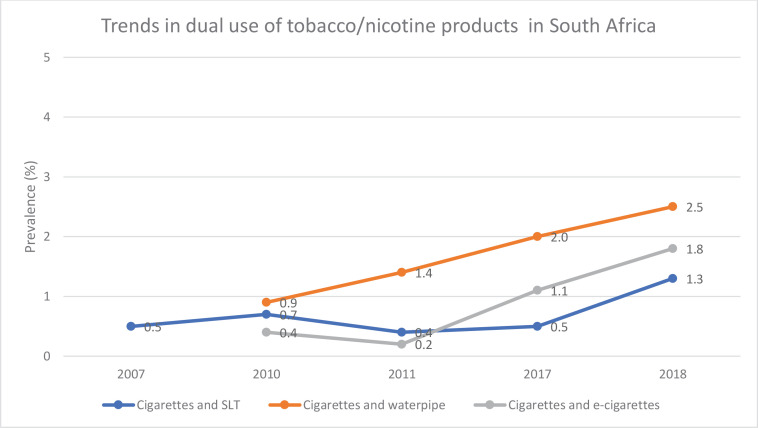
Trends in dual use by tobacco/nicotine product types between 2007 to 2018 in South Africa

Dual use was only assessed for those who used FM cigarettes with the following three products: SLT, waterpipes, or e-cigarettes. Figure 1 shows the trends in the dual use of these products in their respective combinations. The dual use of FM cigarettes with cigars and RYO was not explored due to insufficient data.

*FM Cigarettes and SLT*

The prevalence of the dual use of FM cigarettes and SLT significantly increased from 0.5% (95% CI: 0.3–1.0) in 2007 to 0.7% (95% CI: 0.3–1.3) in 2010 and remained stable at between 0.4% and 0.5% in 2011 and 2017, respectively. However, the prevalence increased to 1.3% (95% CI: 0.8–2.3) in 2018 (p=0.017).

*FM Cigarettes and waterpipe*

The dual use of FM cigarettes and waterpipes could only be calculated from 2010, since no data were collected for waterpipe use in 2007. Prevalence was found to be 0.9% (95% CI: 0.6–1.5) in 2010, and this significantly increased steadily to 2.5% (95% CI: 1.7–3.7) in 2018 (p=0.014) (Table 2).

*FM Cigarettes and e-cigarettes*

The dual use of FM cigarettes and e-cigarettes could only be calculated from 2010 since no data were collected for e-cigarette use in 2007. The prevalence of dual use of FM cigarettes and e-cigarettes was found to be 0.4% (95% CI: 0.2–0.9) in 2010, decreased to 0.2% (95% CI: 0.1–0.5) in 2011, and significantly increased steadily from 1.1% (95% CI: 0.7–1.7) in 2017 to 1.8% (95% CI: 1.0–3.4) in 2018 (p=0.001) (Table 2).

### Change in the use of combustible and any tobacco product (2010–2018)

A mixed-effects logistic regression was conducted for CTP and ATP use to assess the overall change in prevalence over time (2010, 2011, 2017, and 2018), controlling for all demographic characteristics. Also, models were run to assess changes in the prevalence of CTP and ATP use among demographic groups (Table 3). In this second group of models, other demographic characteristics were controlled for (except the one being investigated). The year 2010 was used as a reference category for all models.

**Table 3:** Mixed effects logistic regression of combustible tobacco and any tobacco product use for the years 2011, 2017 and 2018 Ref. 2010^a^

	*Combustible tobacco use^b^*			*Any tobacco product use^c^*		
	*AOR*	*95% CI*	*p*	*AOR*	*95% CI*	*p*
**Overall**						
2011	1.09	0.88–1.34	0.431	1.02	0.84–1.23	0.874
2017	1.08	0.86–1.36	0.510	1.12	0.91–1.37	0.301
2018	1.37	1.06–1.77	**0.018**	1.37	1.08–1.73	**0.009**
**Gender**						
**Male**						
2011	1.21	0.94–1.56	0.134	1.18	0.91–1.52	0.209
2017	1.28	0.97–1.69	0.082	1.28	0.97–1.69	0.083
2018	1.34	0.99– 1.82	0.062	1.39	1.03–1.89	**0.033**
**Female**						
2011	0.82	0.6–1.12	0.209	0.77	0.59–1.02	0.066
2017	0.75	0.51–1.11	0.152	0.91	0.66–1.26	0.584
2018	1.54	1.05–2.26	**0.027**	1.34	0.95–1.88	0.092
**Age** years						
**16–24**						
2011	0.81	0.52–1.27	0.361	0.81	0.52–1.24	0.331
2017	1.36	0.81–2.3	0.246	1.34	0.8–2.24	0.265
2018	0.92	0.55–1.54	0.745	0.9	0.54–1.5	0.699
**25–34**						
2011	1.39	0.93–2.08	0.103	1.33	0.91–1.96	0.146
2017	0.97	0.6–1.57	0.902	1.1	0.69–1.75	0.694
2018	1.81	1.07–3.06	**0.027**	1.85	1.14–3.01	**0.014**
**35–44**						
2011	0.94	0.6–1.46	0.769	0.91	0.6–1.38	0.644
2017	0.63	0.38–1.05	0.076	0.68	0.43–1.1	0.118
2018	1.11	0.64–1.92	0.702	1.11	0.67–1.84	0.685
**45–54**						
2011	1.26	0.81–1.97	0.303	1.27	0.85–1.92	0.243
2017	1.66	0.96–2.87	0.070	1.66	1.02–2.69	**0.042**
2018	1.37	0.81–2.34	0.244	1.76	1.05–2.94	**0.031**
**55–64**						
2011	1.37	0.82–2.31	0.229	0.92	0.57–1.48	0.731
2017	0.96	0.55–1.69	0.893	0.86	0.52–1.42	0.546
2018	1.64	0.97–2.78	0.067	1.37	0.84–2.22	0.205
≥**65**						
2011	0.63	0.32–1.27	0.197	0.76	0.43–1.35	0.353
2017	1.17	0.6–2.31	0.640	1.29	0.76–2.21	0.349
2018	1.89	0.95–3.78	0.071	1.43	0.79–2.59	0.234
**Race**						
**Black African**						
2011	1.12	0.84–1.5	0.442	1.02	0.79–1.32	0.856
2017	1.23	0.9–1.69	0.192	1.24	0.95–1.62	0.112
2018	1.49	1.05–2.11	**0.025**	1.42	1.04–1.93	**0.026**
**Colored**						
2011	1.14	0.8–1.61	0.466	1.1	0.78–1.55	0.575
2017	0.91	0.63–1.3	0.603	0.96	0.67–1.37	0.811
2018	1.61	1.06–2.45	**0.026**	1.62	1.07–2.46	**0.024**
**White**						
2011	0.80	0.52–1.23	0.312	0.83	0.54–1.27	0.394
2017	0.61	0.36–1.03	0.066	0.67	0.4–1.12	0.127
2018	0.89	0.51–1.56	0.680	1.04	0.6–1.8	0.882
**Asian/Indian**						
2011	1.81	0.96–3.42	0.069	1.77	0.93–3.34	0.08
2017	1.68	0.79–3.55	0.178	1.65	0.78–3.48	0.188
2018	1.07	0.55–2.11	0.840	1.11	0.57–2.17	0.763
**Marital status**						
**Married**						
2011	0.93	0.69–1.25	0.626	0.92	0.7–1.21	0.552
2017	0.97	0.68–1.37	0.849	1.09	0.79–1.51	0.61
2018	1.39	0.91–2.12	0.128	1.44	0.98–2.13	0.065
**Widowed/divorce/separated**						
2011	0.98	0.61–1.59	0.945	0.83	0.53–1.28	0.397
2017	1.00	0.55–1.8	0.988	0.96	0.6–1.55	0.883
2018	1.68	0.95–2.95	0.073	1.35	0.85–2.14	0.211
**Never married**						
2011	1.23	0.91–1.65	0.176	1.16	0.87–1.54	0.322
2017	1.15	0.83–1.6	0.393	1.13	0.83–1.55	0.436
2018	1.31	0.92–1.86	0.129	1.31	0.94–1.82	0.114
**Education level**						
**<12 years of schooling**						
2011	1.02	0.78–1.34	0.894	0.92	0.72–1.17	0.489
2017	0.96	0.74–1.25	0.774	0.99	0.79–1.24	0.9
2018	1.19	0.86–1.66	0.294	1.14	0.85–1.54	0.378
**12 years of schooling**						
2011	1.26	0.86–1.85	0.231	1.28	0.88–1.87	0.196
2017	0.13	0.01–1.42	0.095	0.13	0.01–1.44	0.097
2018	1.52	0.97–2.37	0.065	1.63	1.05–2.53	**0.029**
**>12 years of schooling**						
2011	0.93	0.58–1.49	0.767	0.92	0.59–1.45	0.731
2017	0.70	0.13–3.9	0.685	1.36	0.3–6.28	0.692
2018	1.58	0.95–2.63	0.079	1.72	1.06–2.81	**0.03**

AOR: adjusted odds ratio.

a Year 2007 excluded from the analysis due to incomplete data. Year 2010 was used as reference category for all models.

b Combustible tobacco product use: combined cigarette, roll-your-own, cigars and waterpipe tobacco use.

c Any tobacco product includes all combustible and smokeless tobacco products.

* Significant at p<0.05.

*Combustible tobacco use*

Overall, after adjusting for gender, age, race, marital status, and education level, the odds of the prevalence of combustible tobacco use significantly increased by 37% in 2018 (AOR=1.37; 95% CI: 1.06–1.77; p=0.018) compared to 2010. Subgroup analysis for change in the odds of the prevalence by gender shows that the odds of the prevalence for female smokers significantly increased by 54% in 2018 (AOR=1.54; 95% CI: 1.05–2.26; p=0.027) compared to 2010. The odds of the prevalence of combustible tobacco use for participants aged 25–34 years significantly increased by 81% between 2010 and 2018 (AOR=1.81; 95% CI: 1.07–3.06; p=0.027). Moreover, the odds of the prevalence of combustible tobacco use among Black Africans (AOR=1.49; 95% CI: 1.05–2.11; p=0.026) and colored participants (AOR=1.61; 95% CI: 1.06–2.45; p=0.026) significantly increased by 49% and 61%, respectively, over time. However, the prevalence of combustible tobacco use was not significantly associated with marital status or education level, across the years.

*Any tobacco product use*

Overall, the odds of the prevalence of any tobacco product use significantly increased by 37% in 2018 (AOR=1.37; 95% CI: 1.08–1.73; p=0.009) compared to 2010. Furthermore, the odds of the prevalence of any tobacco product use among males (AOR=1.39; 95% CI: 1.03–1.89; p=0.033), those aged 25–34 years (AOR=1.85; 95% CI: 1.14–3.01; p=0.014), 45–54 years (AOR=1.76; 95% CI: 1.05–2.94; p=0.031), Black Africans (AOR=1.42; 95% CI: 1.04–1.93; p=0.026), coloreds (AOR=1.62; 95% CI: 1.07–2.46; p=0.024), and those having 12 years of schooling (AOR=1.63; 95% CI: 1.06–2.81; p=0.030), showed a significant increase between 2010 and 2018. Also, the odds of the prevalence of any tobacco product use among those aged 45–54 years significantly increased by 66% in 2017 (AOR=1.66; 95% CI: 1.02–2.69; p=0.042) and by 76% in 2018 (AOR=1.76; 95% CI: 1.05–2.94; p=0.031) compared to 2010. There was no significant relationship between the prevalence of tobacco product use and marital status.

## DISCUSSION

In the current study, we analyzed self-reported data on prevalence by tobacco or e-cigarette product type and dual use in South Africa during a 12-year period. Our findings suggest an increase in tobacco use in the population studied, particularly in more recent times. The reported use of tobacco and nicotine products increased significantly over time. Tobacco control initiatives and the passing of South Africa’s Tobacco Products Control Act of 1993 (amended in 2008)^17^ may have contributed to reducing tobacco use in the country in the years following the enactment of the law^16^. However, in recent years the gains made over the years are being rolled back^15,16^. Our findings suggest the need to strengthen tobacco control efforts in South Africa.

Further, waterpipe use and e-cigarette use have become popular among the South African population. Though data were not collected for both products in 2007, the gradual increase in the current use of both waterpipes and e-cigarettes from 2010 to 2018, despite their recent introduction into the South African market, is worrisome. This increase could be explained by the marketing of these products with flavorings that mostly appeal to youth^22^. Also, perceptions and beliefs about the harm of these products encourage their popularity, i.e. e-cigarettes have been promoted by manufacturers and some public health advocates as ‘less harmful’ alternatives to tobacco products^23^. Another reason may be that waterpipe tobacco smoking is socially appealing, especially to the youth, because of the flavored tobacco, the attractively colored or sleek devices, and the social atmosphere in which smoking occurs^24^. In addition, there are specialized lounges, bars, and shops for e-cigarettes and waterpipes, which might encourage their use in social settings among young people^6^.

Our results show a lower prevalence compared with some other studies that have recorded a relatively high prevalence of e-cigarette and waterpipe use, especially among targeted young adults in South Africa. For example, Combrink et al.^25^, in their study among Grade 10 students in Johannesburg, found that 60% of participants in their study used waterpipes, which included 20% daily use^25^. Another study by Senkubuge et al.^26^ reported a prevalence of 18.6% among university medical students in Pretoria, South Africa. The differences between our findings and those of these other reports on adolescents and young adults, might be related to the different sampling approach and/or the differences in the populations studied. The current study with prevalence estimates of these products is the only nationally representative sample of South African adults that has been conducted over a 12-year period that we are aware of as of the time of writing this article. Nevertheless, young people are the highest recipients of advertisements^27^, hence it is not a surprise to see youth-focused studies reporting a higher prevalence of use within this population group. Electronic cigarettes remain unregulated in South Africa under the current tobacco legislation, though they are supposed to be subjected to regulations as medicines but are currently promoted as consumer products^28^.

Furthermore, significantly much lower proportions of South Africans use smokeless tobacco compared to cigarettes as reported in our study. A previous study showed that the majority of smokeless tobacco users are Black females of low economic status^29^. However, as with e-cigarettes, the concurrent use of smokeless tobacco products with combustible products remains a public health concern as this increases the risk of contracting tobacco-related diseases^9,30^. Our study did not investigate whether some people who smoke are switching to either of these ‘new’ tobacco/nicotine products.

### Limitations

The current study has several limitations. First, the repeated cross-sectional design of the data using different respondents each year does not lend itself to establishing causal links or explanations for the reported changes in tobacco and nicotine product use patterns found in this study. Additionally, the time intervals between the waves of data collection (2007, 2010, 2011, 2017, 2018) do not provide a consistent linear trend in the patterns of use of these tobacco and nicotine products in the past decade. Further, the measures of tobacco and nicotine product use are self-reported and may not reflect actual use by respondents, as there is potential to provide socially desirable responses that could lead to an underestimation of rates. Regardless of these limitations, this study provides a first report of trends in the dual use of these products using the same survey methods and nationally representative samples. The relatively small percentage of dual users also prevented us from conducting further analysis on the data to further investigate the demographic characteristics of other categories of dual tobacco and nicotine product use. Further research using larger samples would be beneficial.

## CONCLUSIONS

Our findings suggest an increase in the use of all tobacco and nicotine products in the past decade in South Africa. Notably, the dual use of FM cigarettes with either waterpipes, SLT, or e-cigarettes has also increased significantly. Interventions such as the national quitline currently operated by the National Council Against Smoking (NCAS) as well as more accessible tobacco cessation interventions offered within primary healthcare settings to help users quit are needed. Measures to prevent young people from initiating use are also urgently needed to curb the increasing prevalence of tobacco and nicotine products, especially novel and re-emerging products like e-cigarettes and waterpipes. Also, passing the Tobacco Products and Electronic Delivery Systems Control Bill of 2022^31^, which is currently under legislative consideration, will help regulate the advertising and sale of e-cigarettes and waterpipe use, which are not covered by the existing tobacco law. The government should also initiate and sustain public awareness campaigns to continually educate the populace about the increased harms of the use of two or more tobacco/nicotine products as well as nicotine addiction. Future research can explore longitudinal trends in dual use among a cohort.

## Data Availability

Data are available on reasonable request. Requests will be considered on a case-by-case basis.
